# Distribution of metastatic disease in the brain in relation to the hippocampus: a retrospective single-center analysis of 6064 metastases in 632 patients

**DOI:** 10.18632/oncotarget.5828

**Published:** 2015-10-19

**Authors:** San-Gang Wu, Ming-Yue Rao, Juan Zhou, Qin Lin, Zi-Jing Wang, Yong-Xiong Chen, Zhen-Yu He

**Affiliations:** ^1^ Department of Radiation Oncology, Xiamen Cancer Center, the First Affiliated Hospital of Xiamen University, Xiamen, People's Republic of China; ^2^ Department of Radiology, the First Affiliated Hospital of Xiamen University, Xiamen, People's Republic of China; ^3^ Department of Obstetrics and Gynecology, Xiamen Cancer Center, the First Affiliated Hospital of Xiamen University, Xiamen, People's Republic of China; ^4^ Eye Institute of Xiamen University, Fujian Provincial Key Laboratory of Ophthalmology and Visual Science, Medical College of Xiamen University, Xiamen, People's Republic of China; ^5^ Department of Radiation Oncology, Sun Yat-sen University Cancer Center, State Key Laboratory of Oncology in South China, Collaborative Innovation Center of Cancer Medicine, Guangzhou, People's Republic of China

**Keywords:** metastatic brain tumor, hippocampus, whole brain radiation therapy, age

## Abstract

This study aimed to investigate the patterns of brain metastasis and to explore the risk factors affecting hippocampus metastasis (HM). We retrospectively analyzed the clinical information of patients with metastatic disease in the brain. The associations between clinicopathologic variables with HM and peri-hippocampal metastasis (PHM) were evaluated in univariate and multivariate regression analyses. A total of 632 patients with 6064 metastatic lesions were recruited into the present study. Of these, 4.1% (26/632) of patients developed HM, and 5.5% (35/632) of patients developed PHM. Only 0.5% (31/6064) of metastatic lesions were located in the hippocampus and 0.6% (37/6064) were in the PHM. Age ≤60 years was an independent risk factor for HM (odds ratio [OR]: 2.602, 95% confidence interval [CI]: 1.115–6.076, *P* = 0.027) and PHM (OR: 2.555, 95%CI: 1.229–5.310, *P* = 0.012) in univariate and multivariate analyses. The hippocampus is a rare site of brain metastasis. Younger patients (age ≤60 years) had increased risk of developing HM and PHM. The current study provides the opportunity to investigate the clinical feasibility of hippocampal sparing whole brain radiation therapy, especially in older patients.

## INTRODUCTION

Approximately 10% to 80% of patients with malignancies may develop brain metastases from different primary tumor sites [1–4], and there are associated with a dismal prognosis. Whole brain radiotherapy may attenuate the symptoms of brain metastasis and prolong survival time [5]. Moreover, for patients with non-small cell lung cancer (NSCLC) or small cell lung cancer (SCLC), survival can be improved by prophylactic cranial irradiation (PCI) [6, 7]. However, whole brain radiotherapy may cause neurocognitive decline and significantly affect the quality of life (QOL) [8]. Radiation-induced damage to the hippocampus plays a considerable role in the neurocognitive decline of patients after whole brain radiotherapy [9].

The hippocampus, which is a neural structure located in the medial temporal lobe of the brain, has highly sensitive to radiation [10]. Studies have shown that the incidence of hippocampal metastasis (HM) is rarely compared to other sites [11–14], and these results support the hypothesis that hippocampal radiotherapy should be avoided. The Radiation Therapy Oncology Group (RTOG) 0933 trial found that hippocampal sparing whole brain radiation therapy (HS-WBRT) could lessen the effect of radiation on memory [15]. However, not all patients are suitable for HS-WBRT. Currently, an accurate tool to predict hippocampal metastasis is lacked. In this study, we analyzed the distribution of metastatic disease in the brain in patients with malignancies and further explored the factors influencing metastasis to the hippocampus.

## RESULTS

A total of 632 patients met the inclusion criteria were retrospectively analyzed. Table 1 summarized the clinicopathologic characteristics of these patients. The median age at diagnosis was 61 years (range, 20–93 years). There were 395 men (62.5%). NSCLC was found in 64.9% (*n* = 410) of patients, SCLC in 8.0% (*n* = 51) of patients, and breast cancer in 8.9% (*n* = 56) of patients. Moreover, there were 4.1% (26/632) of patients with HM and 5.5% (35/632) with peri-hippocampal metastasis (PHM).

**Table 1:** Clinicopathological characteristics of patients with metastatic brain tumors (*n* = 632)

Characteristics	*n* (%)
Age (years)	
≤60	299 (47.3)
>60	333 (52.7)
Sex	
Male	395 (62.5)
Female	237 (37.5)
Primary site	
Non-small-cell lung cancer	410 (64.9)
Small-cell lung cancer	51 (8.0)
Breast	56 (8.9)
Colorectal	22 (3.4)
Stomach	14 (2.2)
Esophagus	27 (4.3)
Liver	15 (2.4)
Other	37 (5.9)

**Figure 1:**
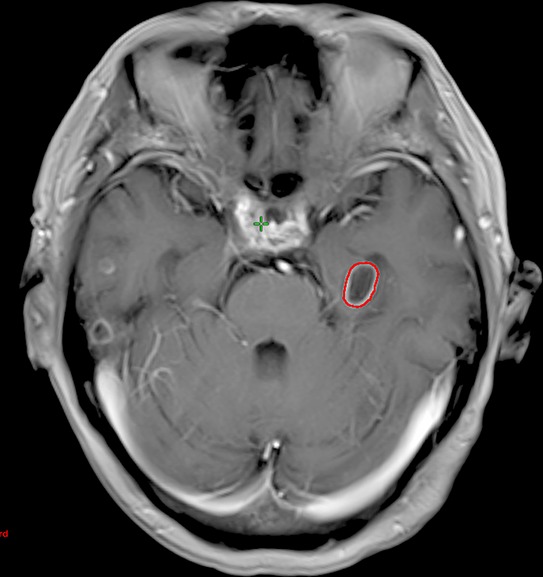
Magnetic resonance image of a patient who has a hippocampal metastasis The red contour represents the hippocampal metastasis.

Among the 632 patients, there were 6064 metastases and the median number of metastases was 3 (range, 1–223). The distribution of metastases is shown in Table 2. The frontal lobe was the most common site of metastases (31.6%, 1919/6064), followed by parietal lobe (18.3%, 1110/6064), cerebellum (18.1%, 1098/6064), occipital lobe (13.0%, 786/6064), temporal lobe (11.7%, 708/6064) and brain stem (6.8%, 412/6064). Thirty-one metastases were within the hippocampus (0.5%, 31/6064) (Figure 1), and 37 metastases were within the peri-hippocampal region (0.6%, 37/6064). The mean hippocampus volume was 4.7 cm^3^ (range, 3.6–6.6 cm^3^). On average, hippocampal avoidance region occupied 2.1% (range, 1.7%–2.9%) of the whole brain volume (Table 3).

**Table 2:** The distribution of metastases by location (*n* = 6064)

Location	*n* (%)
Parietal lobe	1110 (18.3)
Frontal lobe	1919 (31.6)
Temporal lobe*	708 (11.7)
Occipital lobe	786 (13.0)
Cerebellum	1098 (18.1)
Brainstem	412 (6.8)
Hippocampus	31 (0.5)
Total	6064

* Exclusion of metastases involved in hippocampus

**Table 3:** Average volume of hippocampus and hippocampal avoidance regions relateive to the whole brain

Hippocampus volume, cm^3^	Hippocampal avoidance volume, cm^3^	Whole brain volume, cm^3^	Percentage of whole brain occupied by hippocampal avoidance region
4.7 ± 1.0	28.4 ± 4.1	1366.3 ± 160.0	2.1 ± 0.4

The univariate and multivariate logistic regression analysis indicated that age was an independent risk factor for HM and PHM. Patients with age ≤60 years had a higher risk with HM (odds ratio [OR]: 2.602, 95% confidence interval [CI]: 1.115–6.076, *P* = 0.027) and PHM (OR: 2.555, 95% CI: 1.229–5.310, *P* = 0.012) than those with age >60 years (Tables 4 and 5). Of the 26 patients with HM, 18 patients were ≤60 years old. The median number of brain metastases was 19 (range, 2–174), and the median number of brain metastases was 24 (range, 7–47) in 8 patients with age >60 years.

**Table 4:** Univariate logistic regression of hippocampus metastasis and peri hippocampus metastasis

Characteristics	Hippocampus metastasis			Peri hippocampus metastasis		
	OR	95%CI	*P*	OR	95%CI	*P*
Age (years)						
>60	1			1		
≤60	2.602	1.115–6.076	0.027	2.555	1.229–5.310	0.012
Sex						
Male	1			1		
Female	0.686	0.312–1.509	0.349	0.695	0.350–1.379	0.298
Primary site						
NSCLC	1			1		
SLCL	1.646	0.460–5.892	0.444	1.102	0.318–3.820	0.878
Breast	2.582	0.900–7.402	0.078	2.116	0.819–5.470	0.122
Other	0.705	0.201–2.480	0.586	0.636	0.215–1.883	0.413

OR, odds ratio; CI, confidence interval; NSCLC, non-small cell lung cancer; SCLC, small cell lung cancer.

**Table 5:** Multivariate logistic regression of hippocampus metastasis and peri hippocampus metastasis

Characteristics	Hippocampus metastasis			Peri hippocampus metastasis		
	OR	95%CI	*P*	OR	95%CI	*P*
Age (years)						
>60	1			1		
≤60	2.602	1.115–6.076	0.027	2.555	1.229–5.310	0.012
Sex						
Male	1			1		
Female	1.070	0.412–2.778	0.889	1.073	0.478–2.407	0.864
Primary site						
NSCLC	1			1		
SLCL	1.677	0.466–6.041	0.429	1.120	0.321–3.906	0.859
Breast	1.988	0.676–5.849	0.212	1.626	0.615–4.299	0.327
Other	0.723	0.205–2.551	0.641	0.651	0.219–1.935	0.440

OR, odds ratio; CI, confidence interval; NSCLC, non-small cell lung cancer; SCLC, small cell lung cancer.

With potential increased risk of HM (*P* = 0.078) in breast cancer patients compared to NSCLC patients in univariate analysis, therefore, the risk of HM and PHM of breast cancer in comparison with other tumors were analyzed. Among patients with breast cancer, the risk of HM was greater than that in patients with other cancers, but there was no statistical significance (OR: 2.591, 95%CI: 0.938–7.161, *P* = 0.066) and PHM (OR: 2.263, 95%CI: 0.897–5.711, *P* = 0.084), and the results were not affected by the age in breast cancer patients (*P* > 0.05).

## DISCUSSION

In the present study, we retrospectively analyzed 632 patients with 6064 metastases resulting from extracranial malignancies. Our results showed that the incidence of HM and PHM was rarely (4.1% and 5.5%, respectively), and the risk for HM (OR: 2.602) and PHM (OR: 2.555) increased significantly in patients with age ≤60 years.

With advances in imaging and more effective systemic treatments improving survival of cancer patients, the probability of brain metastases maybe potentially increase. PCI may benefit patients with NSCLC or SCLC [6, 7]. However, it is imperative to balance therapeutic efficacy with the risk of radiotherapy-induced damage to the brain. Studies have confirmed that hippocampal radiotherapy may cause neurocognitive decline [8, 9]. In patients receiving brain radiotherapy, the dose of radiation to the hippocampus and temporal lobe may significantly influence cognitive function of patients [19]. The RTOG 0933 trial employed intensity-modulated radiation therapy (IMRT) for HS-WBRT, and the results showed that this technique could significantly improve memory and QOL [15].

In patients with metastatic disease in the brain or those receiving PCI, the decision to perform HS-WBRT is determined by the incidence of HM. Gondi et al. investigated 371 patients with 1133 metastases, and their results showed that 8.6% of patients had PHM, which accounted for 3% of metastases. However, no patient had HM [11]. Harth et al. investigated 100 patients with 856 metastases, and found that 3% patients with HM (0.4% of metastases) [12]. Of 100 patients with 272 metastases, Ghia et al. found that 8% of patients had PHM, which were 3.3% of metastases [13]. In a study of Chinese patients, Wan et al. investigated 488 patients with 2270 metastases, and the results revealed that 1.4% of patients had HM (0.3% of metastases [14]. In the present study, we investigated 632 patients with 6064 metastases, HM and PHM were found in 4.1% and 5.5% of patients, and accounted for 0.5% and 0.6% of metastases, respectively. These percentages are consistent with those previously reported. In addition, no studies have been conducted with Chinese patients with brain metastases to assess the effects of HS-WBRT. Although the present study was retrospective review, it is feasible to implement HS-WBRT for Chinese population. Furthermore, it is now technically and dosimetrically feasible to implement HS-WBRT approaches within clinical practice [15, 16, 22].

In the RTOG 0933 trial, 4.5% of patients developed HM after HS-WBRT [15], while in a study by Oehlke et al., HM recurred in 10% of patients after HS-WBRT [17]. Although there is a potential risk of HM or PHM after HS-WBRT, it is important to develop tools to accurately predict HM or PHM. In a study of SCLC, Kundapur et al. found that the number of metastatic lesions was potentially related to HM (OR: 1.4, 95% CI: 0.9–2.2, *P* = 0.09) [18]. In the study by Oehlke et al., 10% (2/20) of patients developed new HM after HS-WBRT, and new HM occurred concomitantly with multiple other new lesions and not as isolated relapses. However, these lesions were not described in detail [17].

In this study, we found that advancing age was an independent risk factor of HM and PHM. The relationship between HM or PHM and age is unclear. However, studies have found that older age is a poor prognostic factor for survival in patients with metastatic disease in the brain [23–26]. The survival of younger patients with metastatic disease in the brain have a longer life expectancy, and the probability of progression of brain metastases may potentially increase, thereby increasing the probability of HM and PHM. In this study, we found patients with HM are often accompanied by the multiple brain metastases. However, due to the limitations of retrospective studies, we are unable to accurately obtain the time of brain metastases and HM.

Currently, the correlation between specific primary tumor site (including SCLC, NSCLS and melanoma, etc.) and the HM remains unclear [12, 18, 20, 21]. In our study, the risk for HM and PHM in patients with breast cancer was greater than that of patients with other types of cancers, but with no statistical significance. Given that the incidence of HM is very low, it is very difficult to use the type of cancer as to guide to determine whether this procedure is necessary.

There were several limitations to the present study. First, this is a retrospective review from single institution. However, to the best of our knowledge, this study had the largest sample size and our results are similar to other studies. Second, our results are absent the data of HM and PHM after whole brain radiotherapy.

In conclusion, the hippocampus is a rare site of brain metastasis. Patients with younger age had increased risk of developing HM and PHM. The current study provides the opportunity to investigate the clinical feasibility of HS-WBRT, especially in older patients

## PATIENTS AND METHODS

### Patients

Patients were retrospectively reviewed from the First Hospital of Xiamen University between January 2008 and March 2015. These patients were pathologically diagnosed with extracranial malignancies and had brain metastases at initial diagnosis or follow up. The results of magnetic resonance imaging (MRI) including T1-weighted, postcontrast, and axial MRI images were available for all patients. The study protocol was approved by the ethics committee of the First Affiliated Hospital of Xiamen University.

### Delineation of the hippocampus

**Figure 2:**
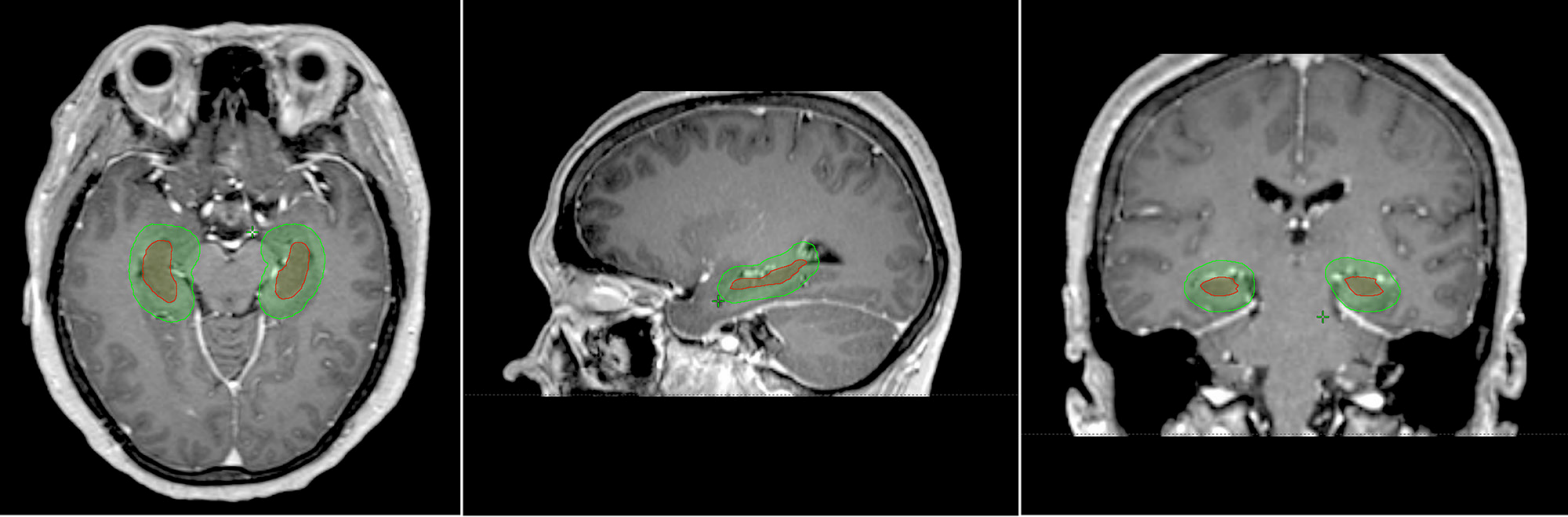
Hippocampus contoured on axial, sagittal, and coronal contrast-enhanced T1 magnetic resonance images The red contour showing the hippocampus contoured and the hippocampal avoidance region contoured in green.

In T1 weighted MRI images, the hippocampus was delineated in accordance with the procedures reported in RTOG 0933. Because of the possibility of errors and displacement during radiotherapy, the methods reported by Gondi et al. and Ghia et al. were also used. The hippocampus plus a 5-mm margin was designated as the peri-hippocampal region (Figure 2) [13, 16]. After delineation of the hippocampus, the distribution of the lesions was documented depending on the location of the lesion from the hippocampus.

### Predictive factors

Age, sex and primary site of malignancy were employed as the risk factors of HM and PHM.

### Statistical analysis

All data were analyzed by using the SPSS statistical software package (version 16.0; IBM Corporation, Armonk, NY, USA). The χ^2^ test and the Fisher's exact probability test was used for categorical variables and analysis of variance for continuous variables to compare the distribution of demographic data among patients with and without HM and PHM. The relationship between patients' characteristics of HM and PHM were examined by univariate and multivariate logistic regression analysis. A *P*-value < 0.05 was considered significant in all analyses.
